# Photodynamic Treatment of Human Breast and Prostate Cancer Cells Using Rose Bengal-Encapsulated Nanoparticles

**DOI:** 10.3390/molecules28196901

**Published:** 2023-10-01

**Authors:** Mir Muhammad Nasir Uddin, Alina Bekmukhametova, Anu Antony, Shital K. Barman, Jessica Houang, Ming J. Wu, James Hook, Laurel George, Richard Wuhrer, Damia Mawad, Daniel Ta, Antonio Lauto

**Affiliations:** 1 School of Science, Western Sydney University, Penrith, NSW 2750, Australia; 2 Department of Pharmacy, Faculty of Biological Sciences, University of Chittagong, Chittagong 4331, Bangladesh; 3 School of Chemistry, University of New South Wales, Sydney, NSW 2052, Australia; 4 Advanced Materials Characterisation Facility, Western Sydney University, Penrith, NSW 2750, Australia; 5 School of Materials Science and Engineering and Australian Centre for NanoMedicine, University of New South Wales, Kensington, NSW 2052, Australia; 6 Biomedical Engineering & Neuroscience Research Group, The MARCS Institute, Western Sydney University, Penrith, NSW 2750, Australia

**Keywords:** tumors, reactive oxygen species, lasers

## Abstract

Cancer, a prominent cause of death, presents treatment challenges, including high dosage requirements, drug resistance, poor tumour penetration and systemic toxicity in traditional chemotherapy. Photodynamic therapy, using photosensitizers like rose bengal (RB) with a green laser, shows promise against breast cancer cells in vitro. However, the hydrophilic RB struggles to efficiently penetrate the tumour site due to the unique clinical microenvironment, aggregating around rather than entering cancer cells. In this study, we have synthesized and characterized RB-encapsulated chitosan nanoparticles with a peak particle size of ~200 nm. These nanoparticles are readily internalized by cells and, in combination with a green laser (λ = 532 nm) killed 94–98% of cultured human breast cancer cells (MCF-7) and prostate cancer cells (PC3) at a low dosage (25 μg/mL RB-nanoparticles, fluence ~126 J/cm^2^, and irradiance ~0.21 W/cm^2^). Furthermore, these nanoparticles are not toxic to cultured human normal breast cells (MCF10A), which opens an avenue for translational applications.

## 1. Introduction

Cancer is one of the leading causes of death worldwide and is a significant impediment to life expectancy. Approximately 10 million people around the globe died from cancer in 2020, with the most common cancers being breast cancer (12.5%), lung cancer (12.2%), colorectal cancer (10.7%), and prostate cancer (7.8%) [1]. Breast cancer is the most common type of cancer in women and is the frontrunner of cancer-related mortality worldwide [2]. In men, prostate cancer remains the second most frequently diagnosed cancer and the fifth most common cause of death globally [3]. However, over the last five years, the death rate has declined, possibly due to the efforts in screening. Nonetheless, the World Health Organization projects that, by 2030, 13.1 million people may die from cancer [4]. Combined with radiotherapy and surgical intervention, chemotherapeutic drugs remain the first line of treatment [5]. However, poor penetration at the tumour site along with systemic and off-target toxicities limit those agents’ use [4]. Photodynamic therapy (PDT) is an established and rapidly developing therapeutic modality in cancer therapeutics due to its noninvasive nature [6,7]. PDT utilises a relatively nontoxic photosensitiser (PS) as a drug to induce the death of cancer cells. These photosensitisers are activated by light, forming reactive oxygen species (ROS) in an excited triplet state which causes highly selective damage to specifically targeted cancer cells but minimal damage to nearby normal cells [8]. Four major photosensitisers are available for PDT, including porphyrin derivatives, porphycenes, chlorin, and phthalocyanine. Activation of these photosensitisers in the excited triplet state occurs through two reaction pathways responsible for the two main mechanisms of PDT-mediated cell death. The Type 1 mechanism results in superoxide anions, hydrogen peroxide, and hydroxyl radicals, while the Type 2 mechanism results in highly reactive singlet oxygen [9]. More oxidative cellular damage is caused by the Type 2 response, which is usually more prevalent [10,11]. Recent developments in PDT are aimed at addressing its key challenges—namely hypoxia and immune tolerance—to enhance its efficacy in treating cancer. Researchers are focusing on the optimisation of PS and ROS, enabling them to function efficiently even in the low-oxygen environments commonly found in tumours. Additionally, there is a growing emphasis on adopting multimodal approaches that synergise PDT with other treatments like chemotherapy or immunotherapy, helping to overcome issues like immune tolerance. These advancements are making PDT increasingly precise and effective, solidifying its role as a promising tool in the fight against various types of cancers [12,13].

Rose bengal (RB) belongs to the xanthene family of fluorescent dyes, such as the very popular fluorescein, that predominantly induces an anticancer effect upon irradiation to produce highly reactive singlet oxygen (Type 2 mechanism). The chlorine and iodine atoms on the RB xanthine ring are responsible for triplet oxygen’s photoreactive conversion to singlet oxygen upon irradiation with green light. A previous study demonstrated that PDT with RB induces its anticancer effect through singlet oxygen-mediated cellular death [8]. RB is a well-known photosensitiser investigated for antibacterial [14], antifungal [15,16], anticancer [17], and tissue-bonding [18] applications. RB and its derivatives in solution exhibited cytotoxic activity against cancer cells [17,19]; for the treatment of hepatocellular cancer, RB in solution has also received approval from the US Food and Drug Administration [20]. However, being a hydrophilic photosensitiser, RB has a limited capacity to cross cellular membranes, therefore restricting its clinical application [21]. Since RB alone has a weak ability for intracellular accumulation, it cannot be used to treat solid tumours [22,23]. Numerous nanoplatforms have been fabricated over the years to overcome this limitation, and nanoparticles have received a great deal of attention among the many nanoplatforms utilised in conjunction with RB [24]. For drug delivery and diagnosis, RB containing gold nanoparticles [25,26,27], upconverting nanoparticles [28] silica nanoparticles [29], aluminium nanoparticles [30], and polymeric nanoparticles [31] are frequently used. Apart from these, nanoparticles have also been used for the codelivery of chemotherapeutic drugs and photosensitisers for synergistic chemo-photodynamic therapy for cancer [32,33].

Chitosan is a biodegradable and biocompatible hydrophilic polysaccharide and has been studied in the synthesis of nanoparticles with sodium tripolyphosphate (TPP) as the crosslinker for drug-delivery applications [34]. Recent developments in chitosan have focused on chemical modifications like acylation, alkylation, and esterification to improve its physical, chemical, and biological properties. These targeted improvements aim to increase chitosan’s solubility, fortify its mechanical strength, improve its drug-release profile, and boost its mucoadhesive properties [35,36]. Chitosan nanoparticles have significant potential as a material for nanoparticle-based anticancer PDT because they can improve drug penetration into tumours [37]. The effectiveness, potency, and safety of numerous chemotherapy medications have been enhanced using chitosan-based nanodrug-delivery systems [35]. In biomedical engineering, chitosan nanoparticles have gained attention for their versatility in drug-delivery systems. Recently, these nanoparticles have been studied for targeted cancer therapy using various approaches, such as encapsulating anticancer drugs, facilitating gene therapy, and implementing surface modifications for specific targeting. These capabilities make chitosan nanoparticles especially valuable for enhancing the effectiveness of different cancer therapies, including chemotherapy, immunotherapy, and PDT [38]. Consequently, there is an ongoing need to optimise their formulation and improve their therapeutic efficacy for clinical translation, particularly in the context of PDT. In the past, RB was covalently crosslinked to free amino groups on the surfaces of chitosan nanoparticles using the EDC crosslinker [N-ethyl-N′-(3-dimethyl aminopropyl) carbodiimide and N-hydroxysuccinimide] [39]. The available literature on RB-encapsulated nanoparticles is exceedingly scarce, even though it has been established that encapsulating the photosensitiser, with, for example, methylene blue [40] or curcumin [41], offers an alternate technique for loading nanoparticles. Encapsulation makes the fabrication process easier since it requires fewer chemical modifications [42]. Here, RB-encapsulated chitosan nanoparticles were synthesized with a peak distribution size of ~200 nm, using our previously established protocol [43]. We successfully tested these nanoparticles on human breast cancer cells (MCF-7) and prostate cancer cells (PC3). We also compared the cytotoxic efficiency of RB-encapsulated chitosan nanoparticles with crosslinked RB and blank chitosan nanoparticles, including ROS generation. Finally, we assessed the dark cytotoxic profile of RB nanoparticles on the cultured normal human breast cells (MCF 10 A) and normal human prostate cells (RWPE-1) to confirm their cytocompatibility.

## 2. Results

### 2.1. Scanning Electron Microscopy and Dynamic Light Scattering

**Figure 1:**
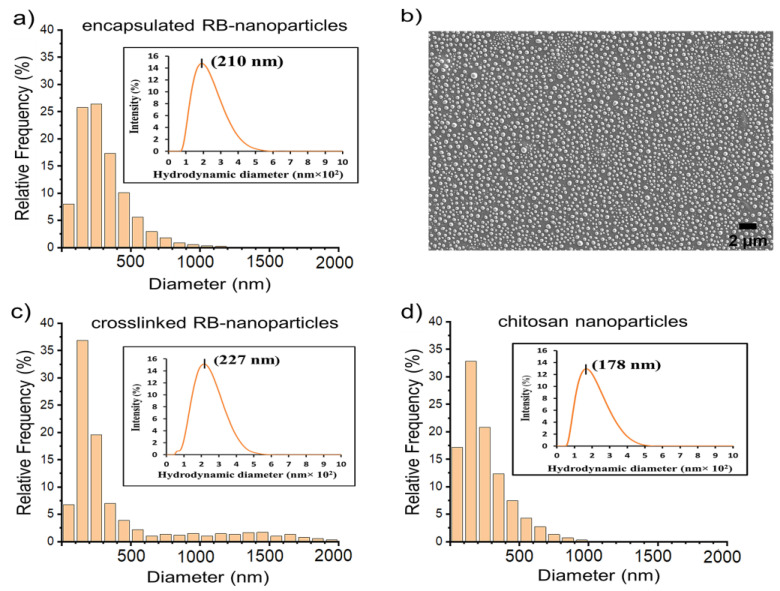
(**a**) Size-distribution histogram of RB-encapsulated nanoparticles, (**b**) SEM image of the same particles; size-distribution histograms of (**c**) RB-crosslinked nanoparticles and (**d**) blank chitosan nanoparticles (without rose bengal). The histograms were generated by following an established protocol and measuring the nanoparticle diameter of three independent sample batches; ~10,000 nanoparticles were measured in each batch. **Insets:** Plots of particle size distributions obtained by DLS. The peak size is indicated in each plot, which is in agreement with the SEM distribution. Three independent DLS experiments were carried out per group.

Particle sizes were analysed with a well-established protocol previously published by our group [43]. The size-distribution histograms of RB-nanoparticles and blank (without rose bengal) chitosan nanoparticles obtained by SEM image analysis are illustrated in Figure 1 and Figure S1 (in Supplementary Materials). All formulations produced nanoparticles with the most frequent size of 100–300 nm and a peak particle size of around 200 nm. DLS confirmed these results, assigning a peak of 210 nm for RB-encapsulated nanoparticles, 227 nm for RB-crosslinked nanoparticles, and 178 nm for blank chitosan nanoparticles (see Figure 1 insets). These nanoparticles also shared comparable polydispersity indices (0.19 to 0.23) and zeta potentials (+21.1 to +25.9) (Table 1).

### 2.2. Photodynamic Treatment of Breast and Prostate Cancer Cells

**Figure 2:**
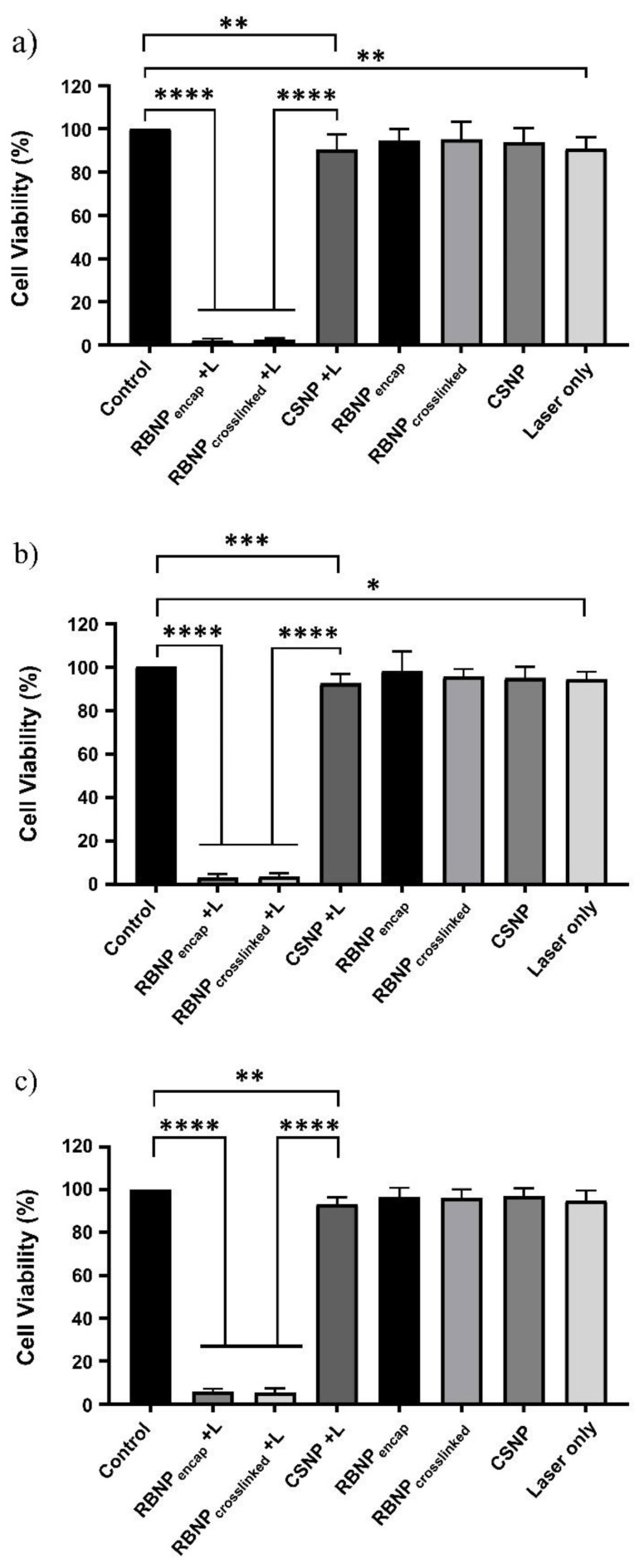
Photodynamic treatment of breast cancer cells using RB-encapsulated or RB-crosslinked nanoparticles. Three different dosage regimens were used in PDT, namely (**a**) 50 μg/mL nanoparticles with 90 mW laser irradiation for 10 min; (**b**) 25 μg/mL nanoparticles with 90 mW irradiation for 10 min; and (**c**) 25 μg/mL nanoparticles with 50 mW irradiation for 10 min. Nanoparticles were incubated with the cells for 1 h. PDT treatments with rose bengal-encapsulated or -crosslinked nanoparticles were very effective for all three dosages, ranging from 3% to 5% cell survival. Unirradiated nanoparticles did not induce significant damage to cancer cells (dark toxicity), while blank chitosan nanoparticles and cells exposed to ‘laser only’ had minor cytotoxicity compared to control cells (~90% cell survival). Three independent experiments were performed in triplicate. Columns with error bars represent mean ± SD. The symbols **** signify *p* < 0.0001, *** *p* < 0.001, ** *p* < 0.01, and * *p* < 0.05 (*p* values determined by one-way ANOVA with Tukey’s post-test).

**Figure 3:**
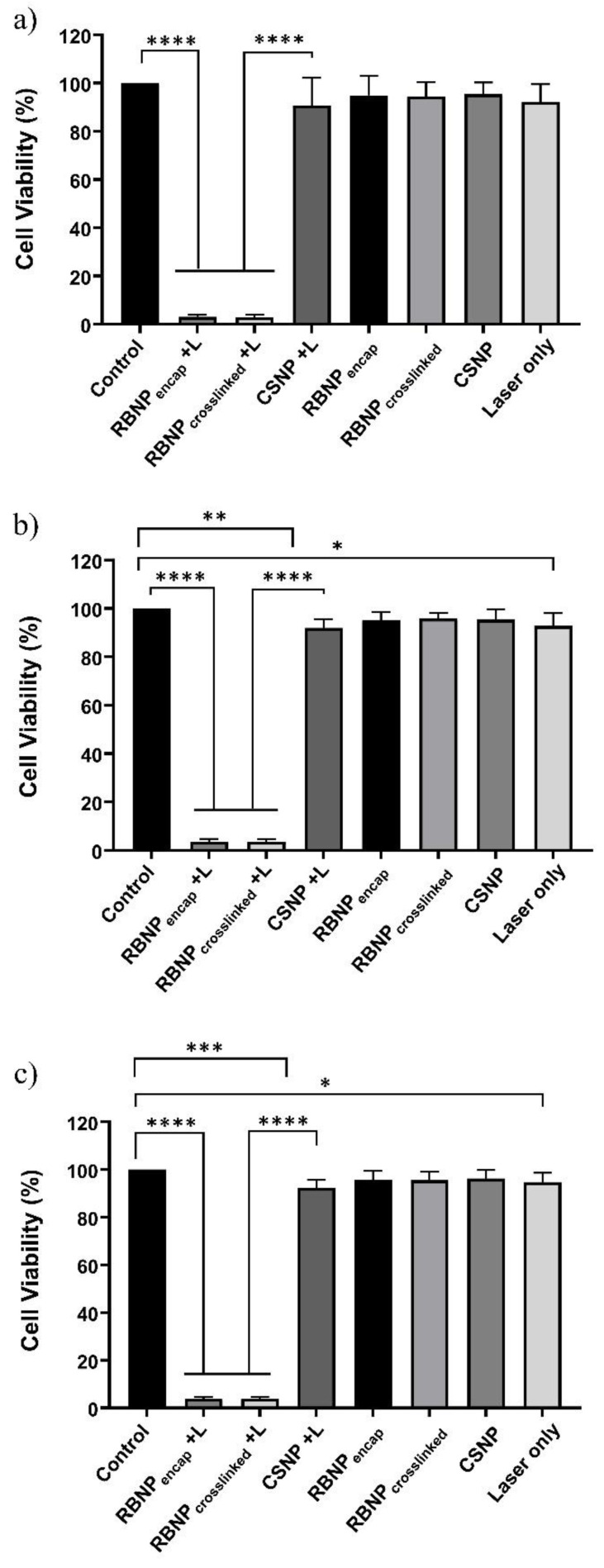
Photodynamic treatment of prostate cancer cells using RB-encapsulated or RB-crosslinked nanoparticles. Three different PDT regimens were used; (**a**) 50 μg/mL nanoparticles with 90 mW irradiation for 10 min; (**b**) 25 μg/mL nanoparticles with 90 mW irradiation for 10 min; and (**c**) 25 μg/mL nanoparticles with 50 mW irradiation for 10 min. Nanoparticles were incubated with cancer cells for 1 h. PDT treatments with rose bengal encapsulated or crosslinked nanoparticles were very effective for all three dosages, ranging from 3% to 4% cell survival. Unirradiated nanoparticles did not induce significant damage to cancer cells (dark toxicity), while blank chitosan nanoparticles and cells exposed to ‘laser only’ had minor cytotoxicity compared to control cells (~90% cell survival). Three independent experiments were performed in triplicate. Columns with error bars represent mean ± SD. The symbols **** signify *p* < 0.0001, *** *p* < 0.001, ** *p* < 0.01, * *p* < 0.05 (*p* values are determined by one-way ANOVA with Tukey’s post-test).

Cancer cells were subjected to three different PDT dosages as follows: (a) 50 μg/mL rose bengal nanoparticles and 90 mW laser power for ten minutes (fluence ~228 J/cm^2^, irradiance ~0.38 W/cm^2^); (b) 25 μg/mL rose bengal nanoparticles and 90 mW laser power for ten minutes (fluence ~228 J/cm^2^, irradiance ~0.38 W/cm^2^); and (c) 25 μg/mL rose bengal nanoparticles and 50 mW laser power for ten minutes (fluence ~126 J/cm^2^, irradiance ~0.21 W/cm^2^). The concentration, e.g., 50 μg/mL, refers to the rose bengal concentration used during the preparation of the rose bengal–chitosan nanoparticles. All experiments were performed in triplicate and each experiment was repeated three times. PDT with encapsulated or crosslinked RB nanoparticles resulted in near-complete eradication of both breast and prostate cancer cells. With regime (a), the cell viability of breast cancer cells was 3 ± 1% and 2 ± 1% for encapsulated and crosslinked nanoparticles, respectively (Figure 2). PDT-treated groups were far more effective than blank chitosan nanoparticles (with no rose bengal) with laser irradiation (90 ± 6% viability) (*p* ˂ 0.0001, one-way ANOVA, Tukey’s post-test). Cell viability was not significantly changed in nonirradiated ‘dark’ incubated blank chitosan or RB nanoparticles when compared to untreated control cells (*p* > 0.05, one-way ANOVA, Tukey’s post-test). Cells treated only with laser irradiation or with chitosan NPs plus a laser exhibited slight cytotoxicity (90 ± 5% and 90 ± 6% viability, respectively). This outcome is nonetheless statistically significant (*p* ˂ 0.01, one-way ANOVA, Tukey’s post-test). The results of regimes (b) and (c) are very similar to the results described for regime (a) in breast cancer cells, as seen in Figure 2. An analogous outcome was observed in prostate cancer cells treated with the three PDT regimes, as shown in Figure 3.

### 2.3. Dark Toxicity in Breast and Prostate Cancer Cells

**Figure 4:**
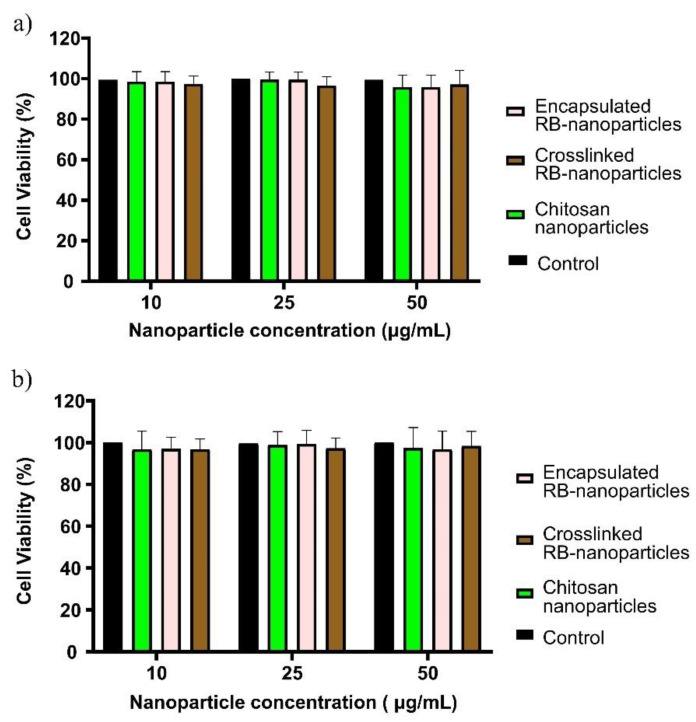
(**a**) Cell viability in breast cancer cells after a 24 h incubation with RB-encapsulated, RB-crosslinked, and blank chitosan nanoparticles at three different concentrations. No significant toxicity occurred when compared to the untreated control cells (*p >* 0.05, 1-way ANOVA, Bonferroni’s multiple comparison test). (**b**) Similar results were observed for prostate cancer cells. Three independent experiments were performed in triplicate.

To explore further the dark toxicity of encapsulated and crosslinked RB nanoparticles, breast and prostate cancer cells were incubated with different concentrations of RB nanoparticles (10, 25, and 50 µg/mL) for 24 h instead of 1 h, as in the previous experiments. RB nanoparticles at different concentrations did not induce any significant toxicity when compared to untreated cells (control) using the MTT assay (*p* > 0.05, one-way ANOVA, Bonferroni’s multiple comparison test, Figure 4a). Blank chitosan nanoparticles (without rose bengal) were also cytocompatible. Similar results were found for prostate cancer cells (Figure 4b).

### 2.4. ROS Measurement and Intracellular Nanoparticle Uptake

**Figure 5:**
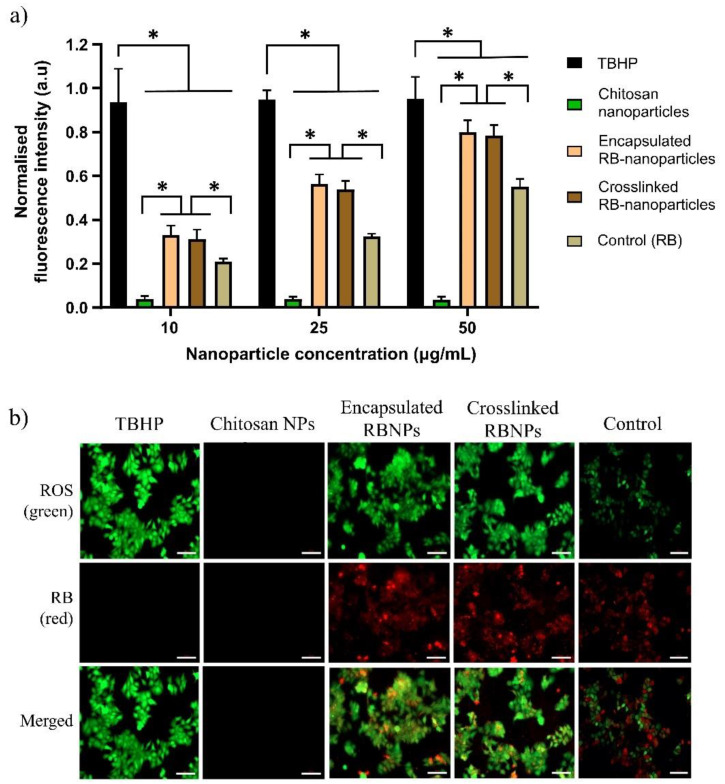
Intracellular ROS generation by RB nanoparticles in breast cancer cells. (**a**) Normalised fluorescence intensity plot of DCFDA probe (excitation = 485 nm, emission = 535 nm) in treated breast cancer cells. No significant difference in ROS generation was found between RB-encapsulated and RB-crosslinked nanoparticles for all treatment groups (*p* > 0.05, 1-way ANOVA, Tukey’s post-test). RB nanoparticles generated more ROS than free RB, which was used as a control (*p* ˂ 0.0001, 1-way ANOVA, Tukey’s post-test). (*) significant at *p*-value ˂ 0.0001 (**b**) Green fluorescence signifies ROS generation detected by the DCFH-DA probe, while the red fluorescence is due to rose bengal nanoparticles. Corresponding images were collected from three independent experiments with representative images depicted.

**Figure 6:**
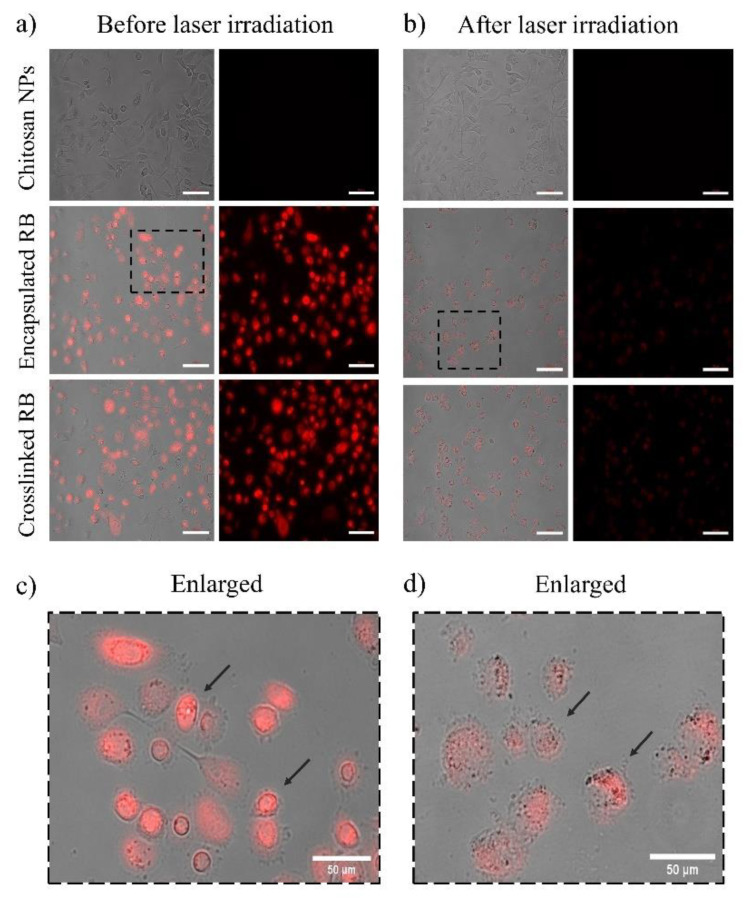
(**a**) Fluorescence microscopic images (right column) show intracellular accumulation of RB-encapsulated nanoparticles, RB-crosslinked nanoparticles and chitosan nanoparticles in prostate cancer cells before laser irradiation. The concentration of both RB-functionalized nanoparticles was 50µg/mL. No fluorescence was observed for chitosan nanoparticles without rose bengal. In the left column, bright field images are overlayed with fluorescence. (**b**) Images of cells after laser irradiation. The RB pigmentation appeared photobleached and the fluorescence was weaker following laser irradiation. The disruption of the cell membrane (black arrows) due to PDT is evident in figures (**c**,**d**) which represent cells before and after laser irradiation, respectively.

DCFDA (2,7-dichlorodihydrofluorescein diacetate) is a cell-permeable redox-sensitive fluorescent probe that is oxidized by ROS to yield the highly fluorescent product DCF (2,7-dichlorofluorescein), the amount of which is directly proportional to the ROS present in the cancer cells. These cells were irradiated with the same parameters used in the PDT experiments (λ = 532 nm, spot size = ~0.5 cm, irradiance = 0.38 W/cm^2^, power = 90 mW, irradiation time = 10 min, and fluence = ~228 J/cm^2^). As shown in Figure 5a, the levels (in arbitrary units) of ROS released at 10 min postirradiation for RB-encapsulated and RB-crosslinked nanoparticles increase with the amount of rose bengal bonded to the nanoparticles. For each rose bengal concentration (10 μg/mL, 25 μg/mL and 50 μg/mL), the measured ROS levels are similar (nonstatistically significant) for encapsulated and crosslinked RB nanoparticles, but significantly higher than that of the rose bengal solution and blank chitosan nanoparticles (without rose bengal). The green fluorescence due to the ROS produced by the laser-irradiated RB nanoparticles is visible, as demonstrated in Figure 5b. These nanoparticles have been extensively internalized by the breast cells during the incubation time, as shown by the red fluorescence of rose bengal. Note that the nanoparticle fabrication protocol ensured that the amount of rose bengal bonded to nanoparticles suspended in solution is similar to the amount of molecular rose bengal dissolved directly in the solution (without nanoparticle carriers). Thus, a comparison between these experimental groups is meaningful. Tert-Butyl hydroperoxide (TBHP) was employed as a positive control over the three concentration groups. Extensive intracellular uptake of RB-encapsulated nanoparticles in prostate cancer cells was also observed, as illustrated in Figure 6.

### 2.5. Dark Toxicity in Noncancerous Human Breast and Prostate Cells

**Figure 7:**
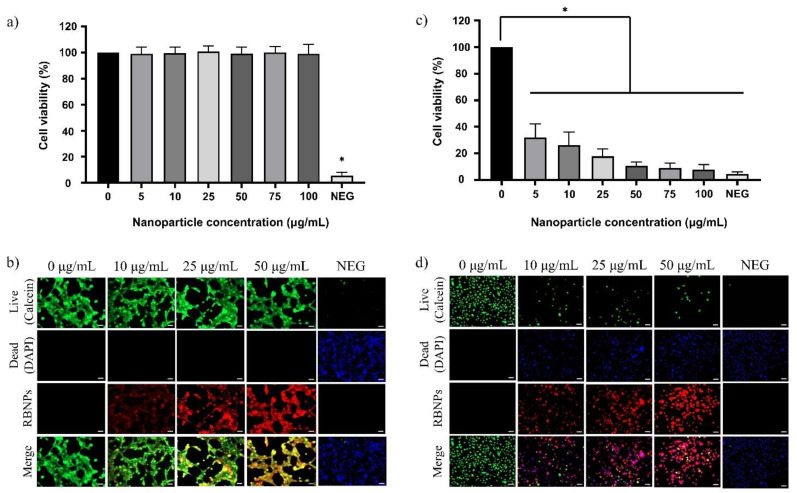
The cytotoxicity of rose bengal chitosan nanoparticles on normal human breast and prostate cells was determined by calculating the percentage of viable cells based on the reduction of MTT compound by metabolically active cells. (**a**) No cytotoxicity was observed in normal human breast epithelial cells at concentrations of 5, 10, 25, 50, 75, and 100 µg/mL. (**b**) The DAPI–Calcein assay confirmed the cytocompatibility of RB nanoparticles in human breast cells. (**c**) A significant decrease in cell viability of normal prostate cells was demonstrated by the MIT assay when RB nanoparticles were incubated at different concentrations. (*) significant at *p*-value ˂ 0.0001 ( one way ANOVA) (**d**) This result is in line with the DAPI–Calcein assay that confirmed the qualitative increase in cell death with increasing nanoparticle concentration. Three independent experiments were performed, and each experiment was done in triplicate.

The effect of encapsulated and crosslinked nanoparticles without laser irradiation on normal (noncancerous) human breast and prostate cells was explored to assess any possible harmful impact on healthy cells surrounding cancer lesions. Since RB-encapsulated nanoparticles contain the same chemical components as crosslinked nanoparticles, apart from the EDC crosslinker, we solely examined the biocompatibility of RB-crosslinked nanoparticles. Human breast cells incubated with different concentrations of RB nanoparticles (5, 10, 25, 50, 75, and 100 µg/mL) for 24 h exhibited no dark cytotoxicity, as there was no significant reduction of cell viability observed (Figure 7a). However, the relative cell viability of the negative control cells, in contrast to the control cells, was significantly lower. RB nanoparticles were also found to be nontoxic by simultaneous labelling of the live–dead cells (qualitative analysis) using the DAPI–Calcein stain (Figure 7b) in breast cells. Green fluorescence was dominant compared to blue in a two-colour fluorescence probe (blue green) in all treatment groups, indicating the healthy cells’ viability. In contrast, green fluorescence in the negative control was substantially decreased. Compared to human breast cells, cultured prostate cells showed a significant reduction in relative cell viability, ranging from 70% to 90%, as the concentration of RB nanoparticles increased from 5 to 100 µg/mL (Figure 7c). The qualitative assessment with DAPI–Calcein stain also supports this observation, as a gradual decrease in green fluorescence was observed compared to blue, with an increasing concentration of nanoparticles (Figure 7d). This is in line with the negative control, which exhibited decreased green fluorescence. The toxicity of RB-crosslinked nanoparticles is most likely due to the presence of rose bengal, considering that blank chitosan nanoparticles induced no significant toxicity to both normal prostate and breast cells (Figure S2, Supplementary Materials).

## 3. Discussion

RB is a powerful source of singlet oxygen (ROS) when combined with green light. When encapsulated within chitosan nanoparticles, ROS release is further enhanced in comparison to RB in solution (control) at similar concentration levels (Figure 5a). Nanoparticles also showed a higher level of intracellular uptake compared to rose bengal in solution, which contributed to the enhanced production of ROS (Figure 5b). Efficient cell uptake and ROS production were the premises for the very effective PDT, which killed 94–98% of breast and prostate cancer cells (Figure 2 and Figure 3). Of note is that blank chitosan nanoparticles produced a very small amount of ROS after irradiation and killed about 10% of cancer cells, confirming that the photo-cytotoxicity was largely due to rose bengal.

**Figure 8:**
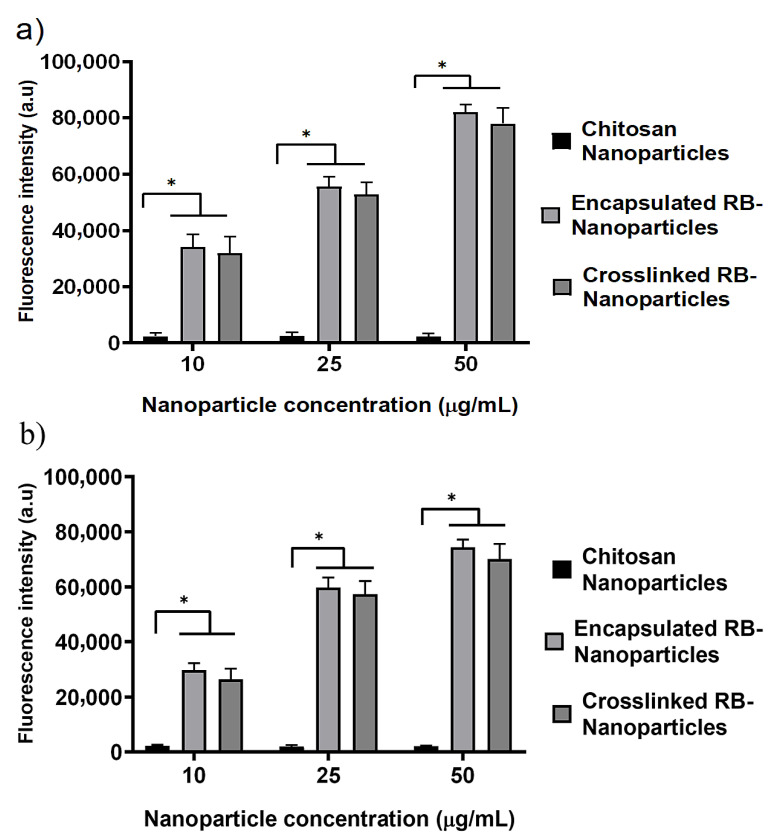
Quantitative intracellular uptake of nanoparticles in (**a**) breast cancer cells and (**b**) prostate cancer cells. Encapsulated and crosslinked nanoparticles have similar levels of cellular uptake at all concentrations. More nanoparticles are internalized in cells as their concentration increases. Blank chitosan nanoparticles without rose bengal do not display significant fluorescence. The fluorescence intensity is given in arbitrary units. (*) significant at *p*-value ˂ 0.0001 (one way ANOVA).

Similar killing rates were also obtained using RB-crosslinked nanoparticles, which displayed no significant difference in cell uptake (Figure 8) and ROS generation (Figure 5a) when compared to RB-encapsulated nanoparticles. The efficacy of our RB-encapsulated nanoparticles is comparable with previous experiments where RB-crosslinked silica nanoparticles, in conjunction with PDT, resulted in the 87% and 94% killing of breast cancer cells (MCF-7) and oral cancer cells (4451), respectively [44]. Furthermore, the results are also comparable to a separate study to reduce cell proliferation in highly aggressive skin cancer cells (SK-MEL-28) using RB-crosslinked mesoporous silica nanoparticles [24]. Despite having similar effectiveness in targeting cancer cells, RB-encapsulated nanoparticles have the advantage of a simpler preparation protocol and the chemical compounds used for their fabrication are the same as those utilized for crosslinked nanoparticles, except for the EDC crosslinker. Although rose bengal has been loaded with nanoparticles for anticancer photodynamic therapy in several studies, it appears that the preferred modality is to crosslink it to the carrier [31], which is usually not chitosan. However, Xie et al. created a pH-responsive nanoparticle by coating silica with encapsulated RB and adding Zeolitic Imidazolate Framework-90 (ZIF-90) as an O_2_ reservoir to combat tumour microenvironment hypoxia [45]. Karthikeyan et al. assessed the use of polyamidoamine dendrimers to encapsulate and deliver RB for PDT of Dalton’s Lymphoma Ascites (DLA) cells [46]. The 20 nm dendrimers encapsulated RB efficiently (92%) and released around 80% within 72 h. Illumination with a Xenon arc lamp (~150 W, 10 min irradiation) generated ROS in the dendrimer–RB complex. Treating DLA cells with this complex and light led to ~75% cell death. Uncertainty remains about whether cells absorbed unbound RB or RB–dendrimers before light exposure. Considering the scarcity of literature on RB-encapsulated nanoparticles, our investigations validate the use of a simple protocol for the synthesis of such particles that are based on chitosan and have a peak size of 200 μm [43,47].

Normal cells are typically impermeable to large molecules due to tight gap junctions on cells; but in cancerous conditions, the junctions become permeable to macromolecules, which allows passive transport of nanoparticles inside cancer cells [48,49,50]. Nanoparticles are currently utilised to deliver cytotoxic drugs to specifically targeted tumours, but their limited penetration and retention sometimes result in clinical ineffectiveness [51]. This unsatisfactory outcome is mainly due to the tendency of larger nanoparticles to disperse around the tumour vasculature rather than entering the tumour interior. In contrast, smaller particles can penetrate more easily but with poor tumour retention [52]. Passive delivery is thought to be most effective when nanoparticles between 100–200 nm are used [53]; our protocol was thus designed to produce nanoparticles with a peak distribution size of ~200 nm. In this study, SEM was employed to quantify approximately 10,000 nanoparticles per sample using our unique method. This method capitalizes on the coffee-ring effect, which aids in the size-based classification of nanoparticles [43]. The nanoparticles were left to dry and distributed on a silicon wafer according to the coffee-ring effect. The distributions obtained from DLS corroborated the SEM findings.

When considering the impact of particle charge, conflicting findings have been reported. It has been shown that, in comparison to neutral and anionic nanoparticles of equivalent size, cationic nanoparticles showed greater penetration in both in vivo tumour models and tumour spheroids [54]. However, neutral nanoparticles have also displayed deeper tissue penetration and distribution within the tumour microenvironment compared to cationic NPs [55]. This may be due to cationic NPs diffusing more slowly and unevenly throughout tumour tissue than neutral nanoparticles, as they tend to form aggregates with oppositely charged components within the tumour matrix [33]. It is still unclear how surface charge influences polymeric nanoparticle transport in the in vivo tumour microenvironment and how it relates to the eventual antitumor effect [55]. However, cancer cells have highly concentrated negatively charged glycoproteins on their surfaces which may play a significant role in their interaction with positively charged nanoparticles and increased permeability [56,57]. Chitosan nanoparticles, in general, carry a positive charge on their surfaces due to the presence of amino groups, which is also evident from the positive zeta potential of our fabricated RB-encapsulated and -crosslinked nanoparticles (25.5 ± 0.4 mV and 22.1 ± 0.7 mV, respectively). There may be a potential benefit to using chitosan nanoparticles for drug delivery in cancer cells due to the uptake of positive nanoparticles into a negatively charged tumour microenvironment [54].

The dark toxicity of RB–chitosan nanoparticles towards normal prostate cells is noteworthy. These cells experienced a significant reduction in relative viability, ranging from 70% to 90%, as the concentration of RB nanoparticles increased from 5 to 100 µg/mL. Rose bengal is likely responsible for this toxicity, considering that blank chitosan nanoparticles are cytocompatible with the same cells (Figure 7). The significance of this cytotoxicity demands careful consideration, and additional studies are needed to examine the potential harm to healthy tissue when translating PDT with RB nanoparticles for prostate cancer treatment.

## 4. Materials and Methods

### 4.1. Materials

Rose bengal (4,5,6,7-tetrachloro-2′,4′,5′,7′-tetraiodofluorescein disodium salt), EDC [N-(3-Dimethylaminopropyl)-N′-ethylcarbodiimide hydrochloride], low molecular weight chitosan (MW = 50–190 kDa, deacetylation degree = 75%), and sodium tripolyphosphate (TPP) were purchased from Sigma-Aldrich (Sydney, Australia). DCFDA/H_2_DCFDA-cellular ROS Assay Kit ab113851 was purchased from Abcam (Cambridge, UK). Glacial acetic acid was sourced from Chem-Supply (Gillman, SA, Australia). NaOH pellets were purchased from PanReac ApplyChem (Barcelona, Spain). All chemicals and reagents were of the highest purity grade commercially available. Deionised water (18.2 MΩ, 25 0 °C) was collected from a Milli-Q Advantage A10 water purification system and used to make sample solutions.

### 4.2. Synthesis of Rose Bengal-Encapsulated Chitosan Nanoparticles

Rose bengal-encapsulated chitosan nanoparticles were synthesised by the ionotropic gelation method, following our original protocol [47]. Briefly, 1 mg/mL of chitosan was dissolved in 1% *v*/*v* acetic acid solution and left stirring for three days. To remove any insoluble macroscopic impurities, the solution was centrifuged (3270 g for 2 h), and the supernatant was recovered for further use. RB (100 μg/mL) was mixed with 0.6 mg/mL TPP; this mixture was added dropwise to the chitosan solution and gently stirred. The entire nanoparticle preparation was kept at room temperature for 30 min with a chitosan: TPP volume ratio of 3:1.

### 4.3. Synthesis of Rose Bengal-Crosslinked Chitosan Nanoparticles

Chitosan nanoparticles were initially synthesised using the following parameters: 5:1 chitosan to TPP mass ratio, pH 5.5 chitosan solution, and 1% *v*/*v* acetic acid [43]. Subsequently, RB was crosslinked to the surface of chitosan nanoparticles using EDC with a standard procedure previously reported by others [39,58]. Briefly, to a flask containing RB (5 mg/mL stock, 100 µg/mL final concentration), the EDC solution (20 mM stock, 2 mM final concentration) was added, giving a final EDC to RB molar ratio of 10:1. The EDC and RB mixture was then added dropwise to the chitosan nanoparticle dispersion and left to stir at room temperature for 3 h.

### 4.4. Purification and Characterisation of Nanoparticles

#### 4.4.1. RB Encapsulation and Crosslinking Efficiency

Nanoparticles are purified by dialysis with a cellulose-membrane bag (molecular weight cutoff of 14 kDa) against Milli-Q water. The details of the purification process were reported in our previous publication [48]. Upon the completion of dialysis, the nanoparticles were ready for photodynamic applications and characterization analysis. A Shimadzu UV-1800 UV-Vis spectrophotometer was used to determine the RB-encapsulation efficiency. RB-functionalised nanoparticles were first centrifuged then the UV-visible spectrum of the supernatant was scanned between 300 and 700 nm to determine the unbound RB concentration.

A standard calibration curve was constructed using RB dissolved in Milli-Q water. The encapsulation efficiency was calculated using the following formula:

Encapsulation Efficiency (EE %) = (C_total_ − C_unbound_)/C_total_ × 100%,

where C_total_ is the initial concentration of RB used in the encapsulation or crosslinking reactions, and C_unbound_ is the concentration of unbound RB in the supernatant after centrifugation.

#### 4.4.2. Dynamic Light Scattering (DLS) and Scanning Electron Microscopy (SEM)

A Zetasizer Nano ZS (Malvern Panalytical Ltd., Malvern, UK) equipped with a He-Ne laser operating at an output power of 4 mW was employed to measure the particle size distribution, hydrodynamic diameter, and zeta potential. Backscattered light was detected at 173° and the intensity-average hydrodynamic diameter was calculated using the Stokes–Einstein equation. The zeta-potential measurements were performed with the same instrument with an applied voltage of 150 V. The obtained data was then processed using Malvern Zetasizer Software (version 7.13) and Microsoft Excel (Version 2308). The analysis of the nanoparticle shape and morphology was performed using a Zeiss Merlin VP Compact field emission gun SEM.

### 4.5. Cell Culture

The complete medium for MCF-7 breast cancer cells (human breast adenocarcinoma, ATCC^®^ HTB-22TM) comprised Dulbecco’s Modified Eagle Medium (DMEM; Thermo Fisher, Melbourne, Australia), 10% (*v*/*v*) fetal bovine serum (FBS; Melbourne, Australia), 1% (*v*/*v*) penicillin/streptomycin, with 10,000 units penicillin and 10 mg streptomycin per ml in 0.9% (*w*/*v*) NaCl (Sigma-Aldrich, Sydney, Australia), and L-glutamine (2 mM and 1% (*v*/*v*)). The complete medium for PC3 prostate cancer cells (human prostate cancer, ATCC^®^ CRL-1435TM) was of the same components, except RPMI 1640 medium (Sigma-Aldrich, Sydney, Australia) was used instead of DMEM. The cells were first cultured in 75 cm^2^ flasks in a 37 °C incubator with 5% CO_2_ until 80% confluence was reached, and then assayed in 96-well plates by seeding 7 × 10^3^ cells/well in triplicate for each treatment. At least two other wells separated the inoculated wells along each row and column (Figure S3, Supplementary Materials). The cells in 96-well plates were cultured for 36 h in the complete medium and then inoculated with nanoparticles and irradiated with the laser; after which, cytotoxicity assays were performed.

### 4.6. Photodynamic Treatment of Breast and Prostate Cancer Cells

#### 4.6.1. Laser System

The photodynamic treatment was delivered by a solid-state laser operating at 532 nm in continuous wave mode. A multimode optical fibre was coupled to the laser with a 200 nm core diameter and 0.22 numerical aperture (CNI Lasers, Changchun, China). The fibre optic was positioned above the plate to obtain a spot size of ~0.24 cm^2^ at the bottom of the well. This setup allowed irradiation of the whole well (Figure S3, Supplementary Materials).

#### 4.6.2. Dosage Regimes

Three different dosage regimes were adopted during the PDT experiments. These were:

1. Fifty μg/mL nanoparticles and 90 mW laser irradiation for ten minutes (Fluence ~228 J/cm^2^, Irradiance ~0.38 W/cm^2^);
2. Twenty-five μg/mL nanoparticles and 90 mW laser irradiation for ten minutes (Fluence ~228 J/cm^2^, Irradiance ~0.38 W/cm^2^);
3. Twenty-five μg/mL nanoparticles and 50 mW laser irradiation for ten minutes (Fluence ~126 J/cm^2^, Irradiance ~0.21 W/cm^2^).

#### 4.6.3. Treatment Groups

MCF-7 and PC3 cancer cells were treated in eight groups, including:

1. PDT + RBNP_crosslinked_ (+RBNP_crosslinked_ + L): the cancer cells were incubated for one hour with RB-crosslinked chitosan nanoparticles, and, then, laser irradiated;
2. PDT + RBNP_encap_ (+RBNP_encap_ + L): the cancer cells were incubated for one hour with RB-encapsulated chitosan nanoparticles, and, then, laser irradiated;
3. Laser + CSNP (+CSNP + L): the cancer cells were incubated for one hour with blank chitosan nanoparticles, and, then, laser irradiated;
4. RB-crosslinked chitosan nanoparticles only (+RBNP_crosslinked_ − L): the cancer cells were incubated for one hour with RB-crosslinked chitosan nanoparticles, but not laser irradiated;
5. RB-encapsulated chitosan nanoparticles only (+RBNP_encap_ − L): the cancer cells were incubated for one hour with RB-encapsulated chitosan nanoparticles, but not laser irradiated;
6. Blank chitosan nanoparticles only (+CSNP − L): the cancer cells were incubated for one hour with blank chitosan nanoparticles, but not laser irradiated;
7. Laser only (–NP + L): the cancer cells were irradiated without using nanoparticles;
8. Control (–NP − L): this consisted of MCF-7 and PC3 cancer cells without treatment.

### 4.7. Cell Viability Assay

An MTT assay was performed to assess the cytotoxic effect of nanoparticles on the viability of the cells [59]. Plates were treated with 50 μL/well of MTT [3-(4,5-dimethyliazol-2-yl)-2,5-diphenyl-2H-tetrazolium bromide] (Thermo Fisher) solution (concentration of 5 mg/mL in PBS). This was added to the existing media in the culture after photodynamic treatment and then incubated for two hours at 37 °C in a 5% CO_2_ environment. The medium was then aspirated and the formazan crystals in each well were solubilised in 100 µL of dimethyl sulfoxide (DMSO; Sigma-Aldrich). The optical absorbance at 600 nm (A600) was measured using a spectrophotometer (Multiskan EX, Thermo Electron, Massachusetts, Waltham, MA, USA) after the plates were gently shaken.

### 4.8. Dark toxicity Measurement

MCF-7 breast and PC3 prostate cancer cells were cultured following the procedure described in Section 4.6. Cells were then incubated with different concentrations of blank chitosan and RB nanoparticles (10, 25 and 50 µg/mL) for 24 h at 37 °C in a humidified atmosphere containing 5% CO_2_. The MTT assay was then used to determine cell viability following the incubation period.

### 4.9. Measurement and Detection of Intracellular Reactive Oxygen Species (ROS)

Intracellular ROS generation by blank chitosan nanoparticles and RB-functionalised nanoparticles was measured with a DCFH-DA probe (DCFDA/H_2_DCFDA—cellular ROS Assay Kit ab113851) using the manufacturer’s protocol. MCF7 cells were cultured and seeded into a 96-well plate which were inoculated with different concentrations of chitosan and RB nanoparticles (10, 25 and 50 µg/mL) for 2 h at 37 °C, then irradiated with a laser for 10 min. The cells were then incubated for 45 min at 37 °C with 20 µM DCFDA reagent (200 µL), and the fluorescence intensity was measured with a BMG POLARstar microplate reader (BMG LABTECH, Ortenberg, Germany). The cells were also imaged to assess nanoparticle-induced intracellular ROS with an inverted Zeiss Axiovert microscope at 20× magnification.

### 4.10. Cytotoxicity Assay

The cultured normal human breast cells MCF10A (ATCC^®^ CRL-10317TM) and normal human prostate epithelial cells RWPE-1 (ATCC^®^ CRL-11609TM) were used to assess the biocompatibility of nanoparticles. MCF10A cells were cultured in the complete medium consisting of 5% (*v*/*v*) horse serum (Thermo Fisher Scientific, Melbourne, Australia), 1% (*v*/*v*) penicillin/streptomycin, cholera toxin (100 ng/mL) (Sigma-Aldrich), and MEGM mammary epithelial cell growth SingleQuotsTM Kit (Lonza, CC-4136) in DMEM/F12 medium (Invitrogen, Mulgrave, Australia). The MEGM SingleQuotsTM Kit provides the medium with human epidermal growth factor (20 ng/mL), hydrocortisone (0.5 mg/mL), and insulin (10 μg/mL). The keratinocyte serum-free medium supplemented with bovine pituitary extract (0.05 mg/mL) and human epidermal growth factor (5 ng/mL) (Thermo Fisher Scientific) was used to culture the normal human prostatic epithelial cells (RWPE-1). The cells were cultured and maintained in the incubator at 37 °C and 5% CO_2_.

#### 4.10.1. Live–Dead Cell Imaging

Calcein-AM and propidium iodide were used to stain normal human breast and prostate cells after blank chitosan nanoparticle treatment. Cells were seeded in black 96-well plates at a cell density of 1 × 10^4^ per well, washed with warm PBS, and incubated for 30 min at 37 °C with a phenol red-free medium containing 5 µM calcein-AM. Cells were then washed and loaded with PI (5 µM)-containing phenol red-free medium, and the fluorescence was captured using an inverted Zeiss Axiovert microscope. Cells treated with calcium ionophore at a concentration of 20 µM were used as a negative control.

Live–dead cell imaging of cultured human breast and prostate cells after treatment with RB nanoparticles was done with DAPI–calcein staining. Normal human breast and prostate cells were cultured and seeded following the procedure detailed in the previous section. After incubating with RB nanoparticles at different concentrations, the cells were gently washed with warm PBS and incubated with serum-free media containing 5 µM calcein-AM. Following this, the cells were washed with warm PBS and incubated with DAPI (0.5 µg/mL)-containing serum-free media for 10 min at 37 °C and 5% CO_2_. An inverted Zeiss Axiovert microscope was used to capture the image.

#### 4.10.2. Cell Viability Assay

An MTT assay was utilised to measure the cell survival and determine the cytotoxicity of RB-crosslinked nanoparticles. This was carried out via the procedure detailed in the previous section. Cell vitality was quantified as a percentage and compared to the mean absorbance of the control cells.

### 4.11. Cellular Uptake and Fluorescence Intensity Quantification of Nanoparticles

Cellular uptake of RB-encapsulated and RB-crosslinked nanoparticles was qualitatively determined by treating the cancer cells with different concentrations of nanoparticles in blacked-walled culture plates. The cells were cultured according to the conditions described in the cell-culture section. Supporting images were captured with an inverted Zeiss Axiovert microscope using a 20× objective.

Quantitative determination of fluorescence intensity was measured with a BMG POLAR star microplate reader (BMG LABTECH, Germany). Following treatment, cells were seeded at a density of 1.5 × 10^4^ cells/well in a 96-well black plate, followed by 36 h of incubation at 37 °C with 5% CO_2_. Cells were then treated with nanoparticles at different concentrations, followed by measurement of fluorescence intensity with the BMG POLAR star microplate reader (excitation: 553 nm, emission: 571 nm).

## 5. Conclusions

Our rose bengal-encapsulated chitosan nanoparticles have a maximum peak diameter of ~200 nm and produce more ROS than free RB in a dose-dependent manner after laser irradiation. These nanoparticles were readily internalised by cells and, in combination with a green laser (λ = 532 nm) killed 94–98% of cultured human breast cancer cells (MCF-7) and prostate cancer cells (PC3) at a low dosage (25 μg/mL RB nanoparticles, fluence ~126 J/cm^2^ and irradiance ~0.21 W/cm^2^). The cytotoxicity study of RB-encapsulated nanoparticles found no significant killing of normal human breast epithelial cells (MCF10A), although some cytotoxicity emerged for normal prostate epithelial cells (RWPE-1). These findings position our encapsulated nanoparticles as a promising candidate for further translational research.

## Figures and Tables

**Table 1:** Summary of nanoparticle properties. DLS was used to measure the diameter, polydispersity index, and zeta potential of nanoparticles. Three independent experiments were performed to obtain the table values that are given as mean ± standard deviation.

Parameters	Rose Bengal-Encapsulated Nanoparticles	Rose Bengal-Crosslinked Nanoparticles	Chitosan Nanoparticles
**pH**	5.4–5.5	5.5–5.6	5.5–5.6
**RB concentration (μg/mL)**	50	50	n/a
**Encapsulation and crosslinking efficiency (%)**	96 ± 3	95 ± 4	n/a
**Charge/zeta potential (mV)**	25.5 ± 0.4	21.1 ± 0.7	25.9 ± 0.9
**Peak maximum (nm)**	210 ± 16	227 ± 21	178 ± 17
**PDI**	0.21 ± 0.02	0.19 ± 0.01	0.23 ± 0.02

## Data Availability

Not applicable.

## Supplementary Materials

The following supporting information can be downloaded at: https://www.mdpi.com/article/10.3390/molecules28196901/s1, Figure S1: SEM images of chitosan and RB-crosslinked nanoparticles, Figure S2: Cytotoxicity of blank chitosan nanoparticles towards noncancerous human breast and prostate cells, and Figure S3: Irradiation assembly for the photodynamic treatment of breast and prostate cancer cells.

## Sample Availability

Samples of the compounds could not be available from the authors upon request.

## References

[1] Sung H., Ferlay J., Siegel R.L., Laversanne M., Soerjomataram I., Jemal A., Bray F. (2021). Global Cancer Statistics 2020: GLOBOCAN Estimates of Incidence and Mortality Worldwide for 36 Cancers in 185 Countries. CA. Cancer J. Clin..

[2] Wilkinson L., Gathani T. (2022). Understanding breast cancer as a global health concern. Br. J. Radiol..

[3] Rawla P. (2019). Epidemiology of Prostate Cancer. World J. Oncol..

[4] Senapati S., Mahanta A.K., Kumar S., Maiti P. (2018). Controlled drug delivery vehicles for cancer treatment and their performance. Signal Transduct. Target. Ther..

[5] Trimble E.L., Ungerleider R.S., Abrams J.A., Kaplan R.S., Feigal E.G., Smith M.A., Carter C.L., Friedman M.A. (1993). Neoadjuvant therapy in cancer treatment. Cancer.

[6] Niculescu A.-G., Grumezescu A.M. (2021). Photodynamic Therapy—An Up-to-Date Review. Appl. Sci..

[7] Li X., Lovell J.F., Yoon J., Chen X. (2020). Clinical development and potential of photothermal and photodynamic therapies for cancer. Nat. Rev. Clin. Oncol..

[8] Panzarini E., Inguscio V., Dini L. (2011). Overview of Cell Death Mechanisms Induced by Rose Bengal Acetate-Photodynamic Therapy. Int. J. Photoenergy.

[9] Li W., Ma Q., Wu E. (2012). Perspectives on the Role of Photodynamic Therapy in the Treatment of Pancreatic Cancer. Int. J. Photoenergy.

[10] Valenzeno D.P. (1987). Photomodification of Biological-Membranes with Emphasis on Singlet Oxygen Mechanisms. Photochem. Photobiol..

[11] Gannon M.J., Brown S.B. (1999). Photodynamic therapy and its applications in gynaecology. Br. J. Obstet. Gynaecol..

[12] Jiang W., Liang M., Lei Q., Li G., Wu S. (2023). The Current Status of Photodynamic Therapy in Cancer Treatment. Cancers.

[13] Zhou Z., Liu Y., Song W., Jiang X., Deng Z., Xiong W., Shen J. (2022). Metabolic reprogramming mediated PD-L1 depression and hypoxia reversion to reactivate tumor therapy. J. Control Release..

[14] Nakonieczna J., Wolnikowska K., Ogonowska P., Neubauer D., Bernat A., Kamysz W. (2018). Rose bengal-mediated photoinactivation of multidrug resistant pseudomonas aeruginosa is enhanced in the presence of antimicrobial peptides. Front. Microbiol..

[15] Amescua G., Arboleda A., Nikpoor N., Durkee H., Relhan N., Aguilar M.C., Flynn H.W., Miller D., Parel J.-M. (2017). Rose bengal photodynamic antimicrobial therapy: A novel treatment for resistant Fusarium keratitis. Cornea.

[16] Houang J., Halliday C., Chen S., Ho C.-H., Bekmukhametova A., Lauto A. (2021). Effective photodynamic treatment of Trichophyton species with Rose Bengal. J. Biophotonics.

[17] Atenco-Cuautle J.C., Delgado-López M.G., Ramos-Garcia R., Ramirez-San-Juan J.C., Ramírez-Ramírez J., Spezzia-Mazzocco T. Rose bengal as a photosensitizer in the photodynamic therapy of breast cancer cell lines. Proceedings of the 17th International Photodynamic Association World Congress.

[18] Lauto A., Mawad D., Barton M., Gupta A., Piller S.C., Hook J. (2010). Photochemical tissue bonding with chitosan adhesive films. Biomed. Eng. Online.

[19] Kim Y.-S., Rubio V., Qi J., Xia R., Shi Z.-Z., Peterson L., Tung C.-H., O’Neill B.E. (2011). Cancer treatment using an optically inert Rose Bengal derivative combined with pulsed focused ultrasound. J. Control. Release.

[20] (2011). Orphan Drug Status for PV-10 for HCC. Oncol. Times.

[21] Chang C.-C., Yang Y.-T., Yang J.-C., Wu H.-D., Tsai T. (2008). Absorption and emission spectral shifts of rose bengal associated with DMPC liposomes. Dye. Pigment..

[22] Wang B., Wang J.-H., Liu Q., Huang H., Chen M., Li K., Li C., Yu X.-F., Chu P.K. (2014). Rose-bengal-conjugated gold nanorods for in vivo photodynamic and photothermal oral cancer therapies. Biomaterials.

[23] Fischer E., Varga F. (1979). Hepatic storage and biliary excretion of rose bengal in the rat. Acta Physiol. Acad. Sci. Hung..

[24] Gianotti E., Martins Estevão B., Cucinotta F., Hioka N., Rizzi M., Renò F., Marchese L. (2014). An efficient rose bengal based nanoplatform for photodynamic therapy. Chemistry.

[25] Cheng Y., Samia A.C., Meyers J.D., Panagopoulos I., Fei B., Burda C. (2008). Highly efficient drug delivery with gold nanoparticle vectors for in vivo photodynamic therapy of cancer. J. Am. Chem. Soc..

[26] Kale M.B. (2022). Rose Bengal-Conjugated Gold Nanoparticles: Quantification of Singlet Oxygen Generation in Photodynamic Therapy. Master’s Thesis.

[27] Cheng Y., Doane T.L., Chuang C.-H., Ziady A., Burda C. (2014). Near infrared light-triggered drug generation and release from gold nanoparticle carriers for photodynamic therapy. Small.

[28] Borodziuk A., Kowalik P., Duda M., Wojciechowski T., Minikayev R., Kalinowska D., Klepka M., Sobczak K., Kłopotowski Ł., Sikora B. (2020). Unmodified Rose Bengal photosensitizer conjugated with NaYF_4_:Yb,Er upconverting nanoparticles for efficient photodynamic therapy. Nanotechnology.

[29] Sabri T., Pawelek P.D., Capobianco J.A. (2018). Dual Activity of Rose Bengal Functionalized to Albumin-Coated Lanthanide-Doped Upconverting Nanoparticles: Targeting and Photodynamic Therapy. ACS Appl. Mater. Interfaces.

[30] Jain A., Koyani R., Muñoz C., Sengar P., Contreras O.E., Juárez P., Hirata G.A. (2018). Magnetic-luminescent cerium-doped gadolinium aluminum garnet nanoparticles for simultaneous imaging and photodynamic therapy of cancer cells. J. Colloid Interface Sci..

[31] Dhaini B., Wagner L., Moinard M., Daouk J., Arnoux P., Schohn H., Schneller P., Acherar S., Hamieh T., Frochot C. (2022). Importance of Rose Bengal Loaded with Nanoparticles for Anti-Cancer Photodynamic Therapy. Pharmaceuticals.

[32] Wang H.-Y., Hou L., Li H.-L., Wang X., Cao Y., Zhang B.-Y., Wang J.-T., Wei S.-J., Dang H.-W., Ran H.-T. (2020). A nanosystem loaded with perfluorohexane and rose bengal coupled upconversion nanoparticles for multimodal imaging and synergetic chemo-photodynamic therapy of cancer. Biomater. Sci..

[33] Zhang Y.-R., Lin R., Li H.-J., He W.-L., Du J.-Z., Wang J. (2019). Strategies to improve tumor penetration of nanomedicines through nanoparticle design. Wiley Interdiscip. Rev. Nanomed. Nanobiotechnol..

[34] Rawal T., Parmar R., Tyagi R.K., Butani S. (2017). Rifampicin loaded chitosan nanoparticle dry powder presents an improved therapeutic approach for alveolar tuberculosis. Colloids Surf. B Biointerfaces.

[35] Virmani T., Kumar G., Sharma A., Pathak K., Akhtar M.S., Afzal O., Altamimi A.S.A. (2023). Amelioration of Cancer Employing Chitosan, Its Derivatives, and Chitosan-Based Nanoparticles: Recent Updates. Polymers.

[36] Zhou Z., Liu Y., Jiang X., Zheng C., Luo W., Xiang X., Qi X., Shen J. (2023). Metformin modified chitosan as a multi-functional adjuvant to enhance cisplatin-based tumor chemotherapy efficacy. Int. J. Biol. Macromol..

[37] Guo H., Li F., Qiu H., Liu J., Qin S., Hou Y., Wang C. (2020). Preparation and Characterization of Chitosan Nanoparticles for Chemotherapy of Melanoma through Enhancing Tumor Penetration. Front. Pharmacol..

[38] Herdiana Y., Wathoni N., Shamsuddin S., Joni I.M., Muchtaridi M. (2021). Chitosan-Based Nanoparticles of Targeted Drug Delivery System in Breast Cancer Treatment. Polymers.

[39] Shrestha A., Hamblin M.R., Kishen A. (2014). Photoactivated rose bengal functionalized chitosan nanoparticles produce antibacterial/biofilm activity and stabilize dentin-collagen. Nanomed. Nanotechnol. Biol. Med..

[40] de Freitas L.M., Calixto G.M.F., Chorilli M., Giusti J.S.M., Bagnato V.S., Soukos N.S., Amiji M.M., Fontana C.R. (2016). Polymeric Nanoparticle-Based Photodynamic Therapy for Chronic Periodontitis In Vivo. Int. J. Mol. Sci..

[41] Sakima V.T., Barbugli P.A., Cerri P.S., Chorilli M., Carmello J.C., Pavarina A.C., Mima E.G. (2018). de O. Antimicrobial Photodynamic Therapy Mediated by Curcumin-Loaded Polymeric Nanoparticles in a Murine Model of Oral Candidiasis. Molecules.

[42] Hermanson G.T. (2013). Microparticles and Nanoparticles. Bioconjugate Techniques.

[43] Bekmukhametova A., Uddin M.M.N., Houang J., Malladi C., George L., Wuhrer R., Barman S.K., Wu M.J., Mawad D., Lauto A. (2022). Fabrication and characterization of chitosan nanoparticles using the coffee-ring effect for photodynamic therapy. Lasers Surg. Med..

[44] Uppal A., Jain B., Gupta P.K., Das K. (2011). Photodynamic action of Rose Bengal silica nanoparticle complex on breast and oral cancer cell lines. Photochem. Photobiol..

[45] Xie Z., Cai X., Sun C., Liang S., Shao S., Huang S., Cheng Z., Pang M., Xing B., Al Kheraif A.A. (2019). O_2_-Loaded pH-Responsive Multifunctional Nanodrug Carrier for Overcoming Hypoxia and Highly Efficient Chemo-Photodynamic Cancer Therapy. Chem. Mater..

[46] Karthikeyan K., Babu A., Kim S.-J., Murugesan R., Jeyasubramanian K. (2011). Enhanced photodynamic efficacy and efficient delivery of Rose Bengal using nanostructured poly(amidoamine) dendrimers: Potential application in photodynamic therapy of cancer. Cancer Nanotechnol..

[47] Bekmukhametova A., Antony A., Halliday C., Chen S., Ho C.-H., Uddin M.M.N., Longo L., Pedrinazzi C., George L., Wuhrer R. (2023). Rose bengal–encapsulated chitosan nanoparticles for the photodynamic treatment of Trichophyton species. Photochem. Photobiol..

[48] Wu J. (2021). The Enhanced Permeability and Retention (EPR) Effect: The Significance of the Concept and Methods to Enhance Its Application. J. Pers. Med..

[49] Alberts B., Johnson A., Lewis J., Raff M., Roberts K., Walter P. (2003). Molecular Biology of the Cell.

[50] Claesson-Welsh L. (2015). Vascular permeability—The essentials. Ups. J. Med. Sci..

[51] Gavas S., Quazi S., Karpiński T.M. (2021). Nanoparticles for Cancer Therapy: Current Progress and Challenges. Nanoscale Res. Lett..

[52] Su Y.-L., Hu S.-H. (2018). Functional Nanoparticles for Tumor Penetration of Therapeutics. Pharmaceutics.

[53] Yu W., Liu R., Zhou Y., Gao H. (2020). Size-Tunable Strategies for a Tumor Targeted Drug Delivery System. ACS Cent. Sci..

[54] Wang H.-X., Zuo Z.-Q., Du J., Wang Y.-C., Sun R., Cao Z.-T., Ye X., Wang J.-L., Leong K., Wang J. (2016). Surface charge critically affects tumor penetration and therapeutic efficacy of cancer nanomedicines. Nano Today.

[55] Jain R.K., Stylianopoulos T. (2010). Delivering nanomedicine to solid tumors. Nat. Rev. Clin. Oncol..

[56] Szasz O. (2013). Essentials of Oncothermia. Conf. Pap. Med..

[57] Cure J.C. (1991). Cancer an electrical phenomenon. Resonant.

[58] Shrestha A., Hamblin M.R., Kishen A. (2012). Characterization of a conjugate between Rose Bengal and chitosan for targeted antibiofilm and tissue stabilization effects as a potential treatment of infected dentin. Antimicrob. Agents Chemother..

[59] Zaman M.S., Johnson A.J., Petersingham G., Muench G.W., Dong Q., Wu M.J. (2019). Protein kinase CK_2_ is involved in zinc homeostasis in breast and prostate cancer cells. BioMetals Int. J. Role Met. Ions Biol. Biochem. Med..

