# Supplementary material for: Photodynamic Treatment of Human Breast and Prostate Cancer Cells Using Rose Bengal-Encapsulated Nanoparticles
Source: Molecules. 2023 Oct 1;28(19):6901. doi: 10.3390/molecules28196901 (PMC10574360; doi:10.3390/molecules28196901)
Supplement: Supplementary file 1 [file molecules-28-06901-s001.zip › molecules-2622895-supplementary.pdf]

Supplementary Materials

Title: Photodynamic treatment of human breast and prostate cancer cells using Rose Bengal-encapsulated nanoparticles

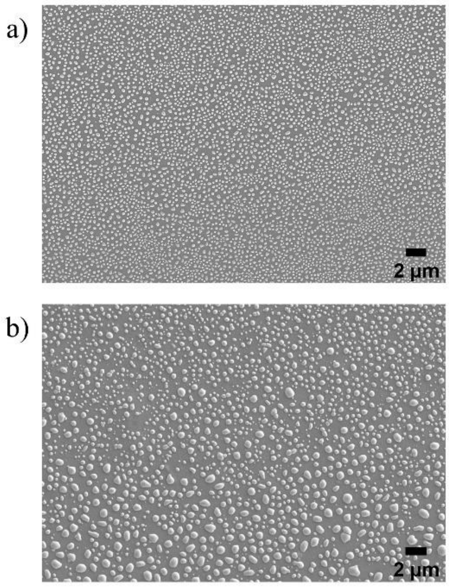

Figure S1. SEM images of the **(a)** chitosan nanoparticles and **(b)** crosslinked RB-nanoparticles (RB = 50 µg/mL). Nanoparticles were prepared with a 5:1 chitosan to TPP mass ratio and chitosan solutions of pH 5.5. Experiments were performed in triplicate.

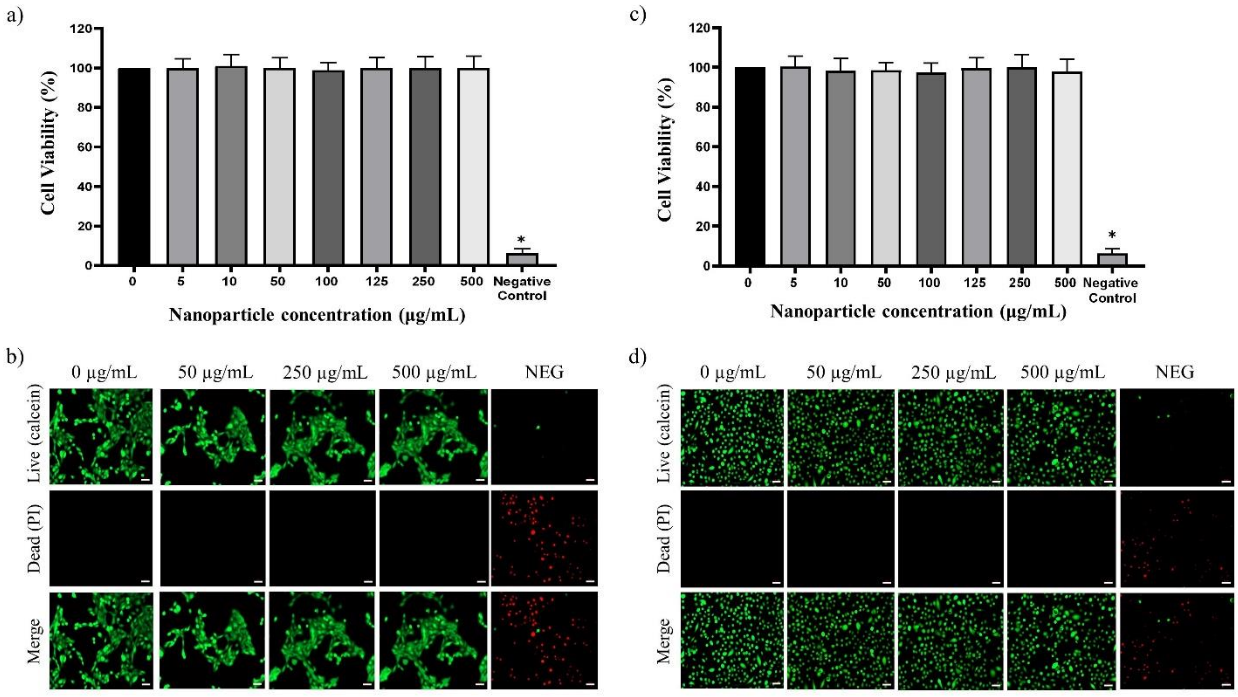

Figure S2. Cytotoxicity studies of blank chitosan nanoparticles (without rose bengal) with non-cancerous human breast and prostate cells were performed by calculating the percentage of viable cells based on the amount of MTT compound that metabolically active cells reduced. Percentage viability of **(a)** human breast cells and **(c)** human prostate cells at nanoparticle concentrations of 5, 10, 25, 50, 100, 125, 250, and 500 µg/mL. Calcein-PI assay confirms the non-toxicity of blank chitosan nanoparticles for **(b)** human normal breast epithelial cells and **(d)** human normal prostate epithelial cells. This is evident in a high number of green (live) cells and a decrease in the number of dead (red) cells in all groups, apart from the negative controls. Data is presented as mean ± SEM, and they reflect the mean of three different experiments that were conducted in triplicates. One-way ANOVA test was used at a significance level of $p = 0.05$.

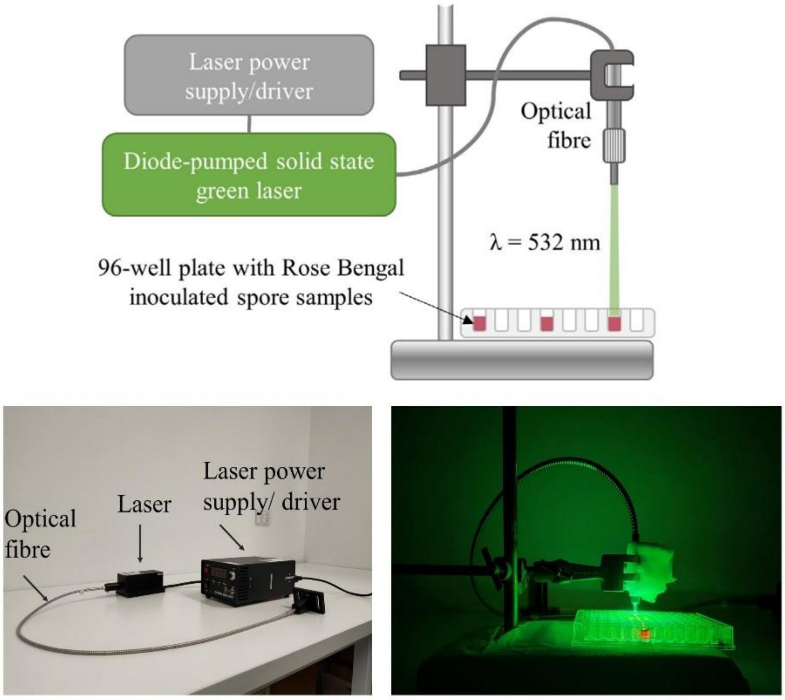

Figure S3. Irradiation assembly for the photodynamic treatment of breast and prostate cancer cells. Cells were seeded into three individual wells (3×200 µL) in a 96-well plate **(top image)**. The laser (λ = 532 nm) had a spot size area similar to the bottom of the well (~0.24 cm^2) to effectively irradiate the cells. Only three wells were used per plate to avoid or minimize over-exposure of coherent light from adjacent wells.
